# Systematic Review of Clinical and Imaging Features for Differentiating Pulmonary Tuberculosis and Nontuberculous Mycobacterial Pulmonary Diseases

**DOI:** 10.7759/cureus.97616

**Published:** 2025-11-23

**Authors:** Akihiko Goto, Ishikawa Kentaro, Taisei Hirakawa, Kosaku Komiya

**Affiliations:** 1 Department of Respiratory Medicine, Oita Medical Center, Oita, JPN; 2 Department of Respiratory Medicine and Infectious Diseases, Faculty of Medicine, Oita University, Oita, JPN

**Keywords:** bronchiectasis, differential diagnosis, interferon-gamma release assay (igra), nontuberculous mycobacteria (ntm), tuberculosis

## Abstract

Pulmonary tuberculosis (TB) and nontuberculous mycobacterial pulmonary disease (NTM-PD) often present with similar clinical and radiological features, making accurate differentiation essential. This systematic review aimed to summarize diagnostic features that distinguish pulmonary TB from NTM-PD. A systematic review following Preferred Reporting Items for Systematic Reviews and Meta-Analyses guidelines searched PubMed for studies differentiating pulmonary TB from NTM-PD. Eligible studies were screened by pulmonary disease specialists, and data on study design, demographics, and diagnostic factors were extracted. Risk of bias was assessed using the Quality Assessment of Diagnostic Accuracy Studies-2 tool, and meta-analysis with random-effects models evaluated effect sizes and heterogeneity. A PubMed search identified 847 studies, of which 11 observational studies met the inclusion criteria. Imaging findings were the most commonly distinguishing features, with the absence of bronchiectasis more frequently observed in pulmonary TB. Meta-analysis showed a significant association between the absence of bronchiectasis and TB diagnosis (odds ratio (OR): 0.19; 95% CI: 0.10-0.36; I² = 82%). Three studies also reported that positive interferon-gamma release assay (IGRA) results were significantly associated with pulmonary TB (OR: 17.34; 95% CI: 6.71-44.75; I² = 68%). In addition, pulmonary TB was more frequently associated with upper lobe opacities and less involvement of the right middle lobe than NTM-PD. These findings reveal that radiological patterns and the IGRA results may serve as useful markers for differentiating pulmonary TB and NTM-PD. Further large-scale studies are required to validate these findings.

## Introduction and background

Although the global incidence of tuberculosis (TB) is gradually declining, the disease remains prevalent in certain regions, especially in Southeast Asia, South Asia, and Africa, highlighting the need for continued prevention efforts [1]. By contrast, the incidence of nontuberculous mycobacterial pulmonary disease (NTM-PD), a lung condition owing to acid-fast bacteria, is increasing. Between 2008 and 2015, the annual incidence of NTM lung disease in North America increased from 3.13 to 4.73 cases per 100,000 person-years, with similar trends reported in other regions [2,3]. Unlike pulmonary TB, which is transmitted from person to person via airborne droplets, NTM is typically not transmitted between individuals [4]. Therefore, early diagnosis and infection control are crucial for TB to prevent transmission, whereas such measures are less urgent for NTM.

Moreover, although pulmonary TB is treated with a standardized regimen of multiple anti-TB drugs, NTM-PD is required to be managed using an individualized approach based on the specific bacterial strain, often involving drug regimens that differ from those used for TB. Therefore, accurate diagnosis is critical from clinical and public health perspectives [4]. Pulmonary TB typically presents with symptoms such as cough, sputum production, and fever, along with radiological findings, including lobular central nodular opacities and cavitary lesions [5,6]. By contrast, NTM-PD presents with similar symptoms, such as sputum production, cough, and fatigue, and overlapping radiological features including nodular opacities, bronchiectasis, and cavitary lesions [7]. Although several studies have investigated differences in clinical symptoms and laboratory findings between these two diseases [8,9], no comprehensive review has yet synthesized these findings. This systematic review aims to consolidate the existing evidence to aid in the differential diagnosis of pulmonary TB and NTM-PD.

## Review

Methods

Search Strategy and Selection Criteria

This systematic review was conducted in accordance with the Preferred Reporting Items for Systematic Reviews and Meta-Analyses guidelines [10]. A comprehensive literature search was performed using the PubMed database to identify studies that addressed the differentiation between TB and NTM-PD. The search terms included (tuberculosis) AND (nontuberculous OR non-tuberculous) AND (differential) AND (diagnosis) (assessed on February 13, 2025). No publication date or text availability, or article type restrictions were applied.

Exclusion criteria were as follows: non-English publications, studies without abstracts, review articles, case reports, and those not focused on differentiating pulmonary TB from NTM-PD (mismatched sample). Studies that examined unrelated factors rather than clinical or laboratory findings (mismatched outcome) were also excluded.

Data Collection and Outcomes

Two pulmonary disease specialists (KI and AG, with 3 and 15 years of experience, respectively) independently screened the titles, abstracts, and full texts of the retrieved articles. Any disagreements were resolved through discussions with a third reviewer (KK, a clinical researcher with 21 years of experience). From each included study, the following data were extracted: study design, sample size, patient age, and factors associated with common findings in patients with pulmonary TB and NTM-PD.

The primary outcome of this study was to identify clinical, radiological, and laboratory factors that distinguish pulmonary TB from NTM-PD. The secondary outcome was to assess the methodological quality and risk of bias of the included studies.

Risk of Bias Assessment

The risk of bias was evaluated using the Quality Assessment of Diagnostic Accuracy Studies-2 (QUADAS-2) tool, following Cochrane Handbook for Systematic Reviews of Diagnostic Test Accuracy [11]. This tool assesses four domains: patient selection, index test, reference standard, and flow/timing. Each domain is evaluated for risk of bias, and the first three domains are assessed for concerns regarding applicability. The QUADAS-2 tool is widely accepted for enhancing methodological transparency in bias assessment.

Statistical Analysis

Meta-analysis was performed using EZR (Saitama Medical Center, Jichi Medical University, Saitama, Japan), a graphical user interface for R (version 4.3.1; The R Foundation for Statistical Computing, Vienna, Austria) [12]. A forest plot was generated to visualize the effect sizes and their corresponding 95% confidence intervals (CIs). Statistical heterogeneity was assessed using Higgins’ I² statistic. A random effects model was applied when considerable heterogeneity was detected; otherwise, a common effect model was used for comparison. Moreover, funnel plots were constructed and visually inspected for asymmetry to evaluate publication bias. All statistical tests were two-tailed, and a p-value <0.05 was considered statistically significant.

Results

Database Search and Characteristics of the Included Studies

**Figure 1:**
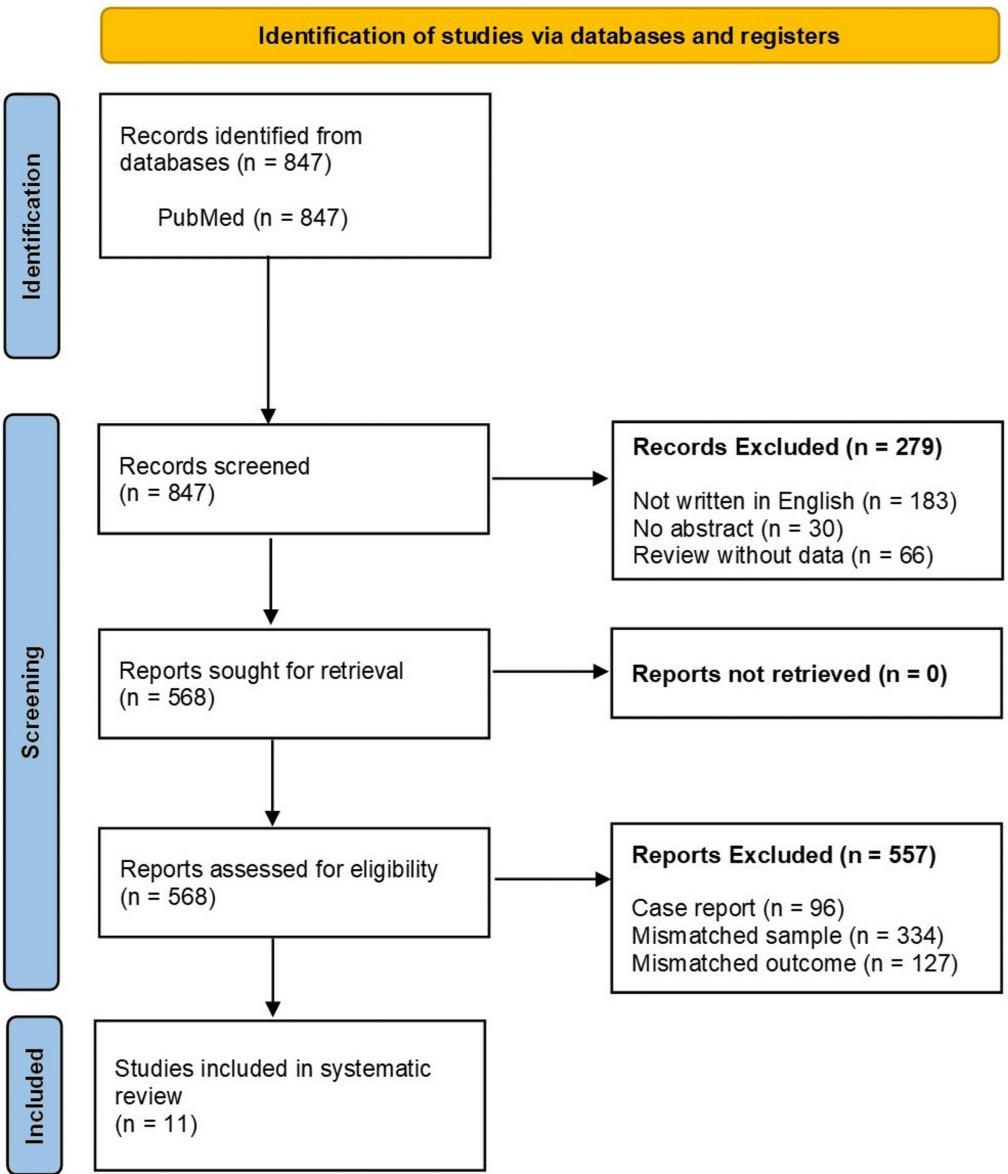
Preferred Reporting Items for Systematic Reviews and Meta-Analyses (PRISMA) flow diagram illustrating the selection process of eligible studies.

In total, 847 studies were identified through the PubMed database search using the predefined criteria. Following screening, 836 studies were excluded for not meeting the inclusion criteria (Figure 1). Consequently, the remaining 11 observational studies were included in this systematic review [8,9,13-21], which reported clinical or diagnostic features more commonly associated with pulmonary TB than with NTM-PD (Table 1). Among the 11 studies, seven were retrospective, and four were prospective in design. Imaging findings emerged as the most frequently reported differentiators, with specific emphasis on the absence of bronchiectasis. In addition, four studies highlighted blood sample results as differential indicators, whereas three studies emphasized symptom-based differences.

**Table 1:** Characteristics of the included studies. COPD: chronic obstructive pulmonary disease, IGRA: interferon-gamma release assay, IQR: interquartile range, NTM: nontuberculous mycobacteria, QFT: QuantiFERON, SD: standard deviation, TB: tuberculosis

Author, Year	Country	Study design	Sample size	Participants’ age (years)	Key findings in patients with pulmonary tuberculosis
BaHammam, 2005 [8]	Saudi Arabia	Retrospective	TB 216, NTM 70	Not described	Weight loss, fever, hilar adenopathy, absence of bronchiectasis
Kobashi, 2006 [12]	Japan	Prospective	TB 50, NTM 100	TB: mean 60.4 (SD 10.0); NTM: mean 64.4 (SD 10.2)	Positive QFT result
Theerawit, 2010 [13]	Thailand	Retrospective	TB 63, NTM 17	TB: mean 46.9; NTM: mean 51.2	Symptom resolution after 5 months of treatment, absence of middle and lower lobe involvement
Yuan, 2014 [14]	Taiwan	Prospective	TB 75, NTM 20	TB: mean 65.6 (SD 18.2); NTM: mean 67.6 (SD 14.6)	Nodules, absence of bronchiectasis, absence of cystic changes
Kim, 2014 [15]	Korea	Retrospective	TB 112, NTM 30	TB: median 49 (IQR 41–61); NTM: median 62 (IQR 50–72)	Younger age (<65 years), treatment naïve for TB
Chu, 2015 [16]	China	Prospective	TB 210, NTM 124	TB: mean 54; NTM: mean 59	Thick cavities (diameter <3 cm), nodule (diameter <1 cm), atelectasis, mediastinal lymph nodes, lymph node calcification, absence of bronchiectasis
Chen, 2021 [17]	China	Retrospective	TB 61, NTM 64	TB: mean 56.3 (SD 17.0); NTM: mean 58.6 (SD 11.7)	Male sex, positive QFT result, absence of bronchiectasis, absence of right middle lobe lesion
Xu, 2022 [18]	China	Retrospective	TB 200, NTM 200	TB: mean 50.3 (SD 11.8); NTM: mean 50.6 (SD 12.1)	No comorbid lung disease, absence of cough and hemoptysis, thick-walled cavities, lung consolidation, atelectasis, lung destruction, reduced lung volume, calcification (pulmonary and lymph node), acinar nodules, pleural thickening/effusion, absence of bronchiectasis and centrilobular nodules
Sanogo, 2023 [19]	Mali	Prospective	TB 32, NTM 58	All: mean 42.4 (SD 13.7)	Elevated basophil count, low absolute eosinophil count, increased platelet count
Liu, 2023 [20]	China	Retrospective	TB 101, NTM 97	TB: mean 44.1 (SD 19.4); NTM: mean 58.2 (SD 14.6)	Positive Wantai TB-IGRA result, lymph node calcification, pleural effusion, hilar and mediastinal lymphadenopathy, absence of bronchiectasis
Zhang, 2024 [9]	China	Retrospective	TB 103, NTM 103	TB: median 55.5 (IQR 32.0–67.5); NTM: median 66 (IQR 57–73)	Diabetes, absence of COPD, absence of bronchiectasis, absence of lung cavities

Risk of Bias Assessment

**Figure 2:**
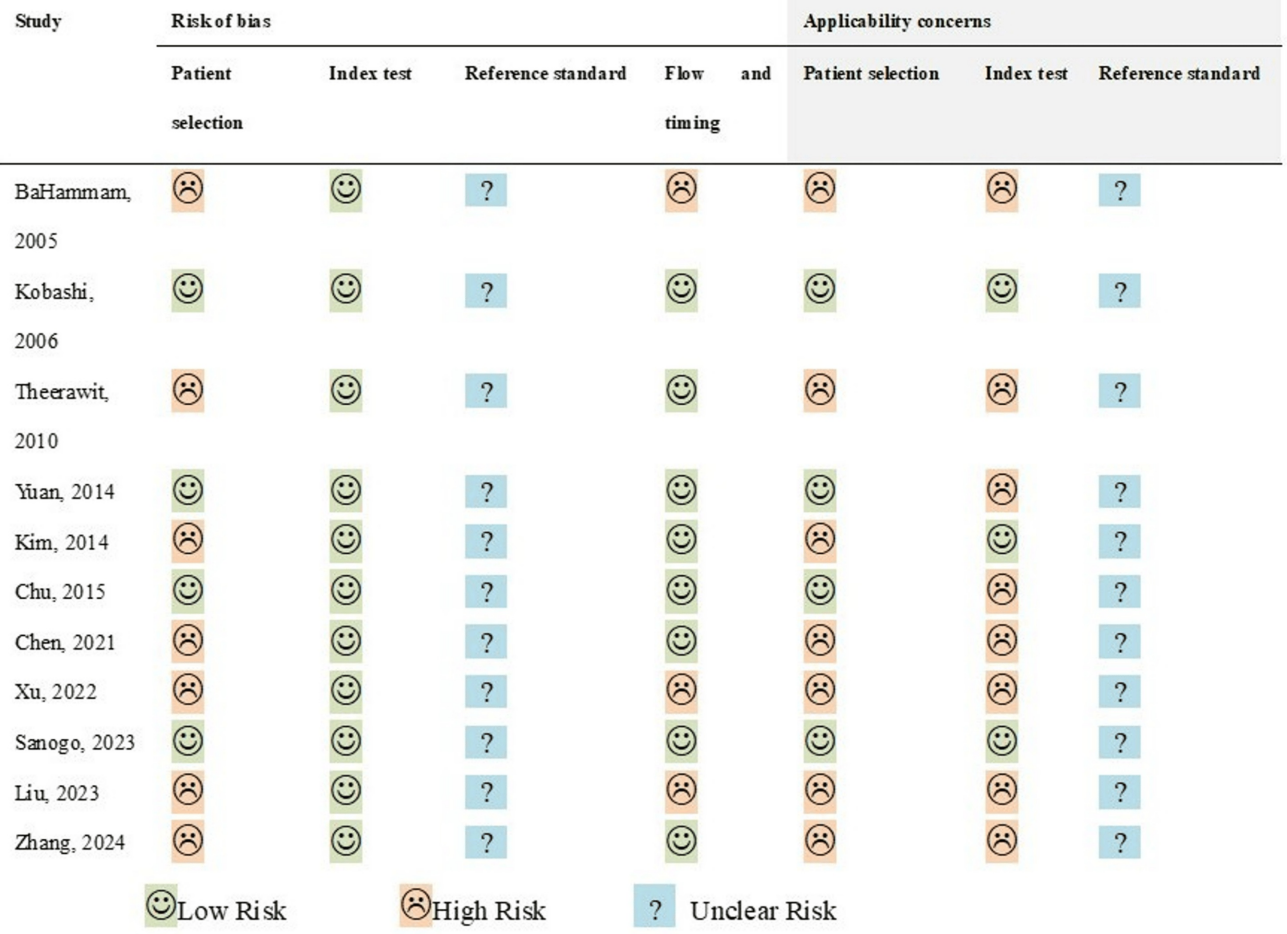
Quality assessment of observational studies using the Quality Assessment of Diagnostic Accuracy Studies-2 tool.

The risk of bias assessments is summarized in Figure 2. Overall, study quality in the “index Test” domain was high. However, under “Applicability Concerns,” the quality was lower because of the subjective interpretation of chest imaging results by the study authors. Similarly, the “Patient Selection” domain scored relatively low, primarily because most studies employed a retrospective design. None of the studies explicitly adhered to a standardized reference for confirming diagnoses; thus, the “reference Standard” domain was rated as having an “Unclear Risk.”

Bronchiectasis

**Figure 3:**
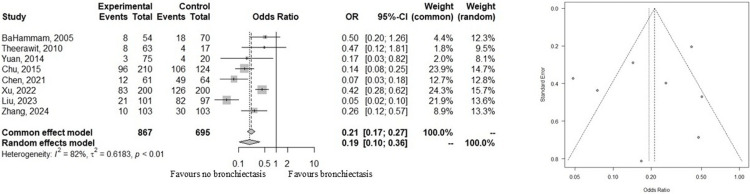
Forest and funnel plots comparing the presence and absence of bronchiectasis in patients diagnosed with pulmonary tuberculosis.

Eight of the 11 studies reported that bronchiectasis was less commonly observed in patients with pulmonary TB. Meta-analysis revealed a significant association between the absence of bronchiectasis and TB diagnosis, despite high heterogeneity across studies (odds ratio (random-effects model): 0.19; 95% CI: 0.10-0.36; I² = 82%), as shown in Figure 3. Although the number of contributing studies was limited, funnel plot symmetry suggests a low risk of publication bias.

Distribution of Pulmonary Opacities

The distribution of pulmonary opacities was discussed in two studies. One study reported that, compared with NTM-PD, pulmonary TB exhibited a predominance of opacities in the upper lobe [14]. Another study reported that opacities in the right middle lobe were less frequently observed in pulmonary TB cases [18].

*Interferon-Gamma Release Assay (* *IGRA* *) Results*

**Figure 4:**
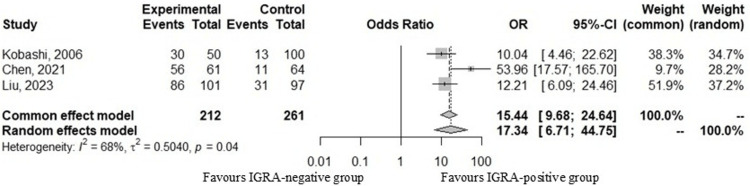
Forest plots comparing positive and negative IGRA results in patients diagnosed with pulmonary tuberculosis.

Three studies indicated that positive IGRA results were more frequently observed in patients with pulmonary TB. Meta-analysis demonstrated a significant association between positive IGRA results and TB diagnosis, with moderate heterogeneity (odds ratio (random-effects model): 17.34; 95% CI: 6.71-44.75; I² = 68%), as shown in Figure 4.

Discussion

This study demonstrated that the absence of bronchiectasis and positive IGRA results were particularly valuable for differentiating pulmonary TB from NTM-PD. However, the findings also suggest that clinical symptoms alone are insufficient for reliably differentiating between the two diseases.

In the present meta-analysis, the absence of bronchiectasis was substantially associated with the diagnosis of pulmonary TB, which typically manifests with cavitary lesions predominantly in the upper lobes, limited chronic airway destruction, and may include calcification, all factors that could suppress bronchial dilation [22,23]. By contrast, bronchiectasis, often resulting from chronic airway inflammation, has been reported in up to 90% of NTM-PD cases, underscoring a notable difference in its prevalence between TB and NTM-PD [24].

The underlying mechanisms of airway inflammation also differ between the two diseases. TB is primarily characterized by the formation of necrotizing granulomas, which result from host immune responses [25]. On the other hand, NTM-PD is associated with the persistent activation of macrophages and neutrophils, destroying bronchial cartilage and smooth muscle layers, ultimately resulting in irreversible bronchiectasis [26]. These pathophysiological differences are reflected in the distinct radiological findings commonly observed in each disease.

Our analysis revealed that the majority of pulmonary opacities in TB were predominantly located in the upper lobe rather than in the right middle lobe. These findings are consistent with previous studies indicating that pulmonary TB is commonly associated with nodular and cavitary lesions in the upper lobes, whereas NTM-PD tends to present with nodular opacities and bronchiectasis in the right middle lobe and left lingular segment [27]. Several mechanisms have been proposed to explain the upper lobe predominance in TB, including the higher oxygen tension in the upper lobes, favoring the growth of *M. tuberculosis*, and the preferential transport of tubercle bacilli to the upper lobes via the thoracic duct [28]. By contrast, poor airway clearance in the right lobe and left lingular segment [29] may contribute to the localization of NTM-PD lesions in these areas. Therefore, the distribution of pulmonary opacities, especially in the upper lobe or right middle lobe, may serve as a useful diagnostic indicator.

A significant association was also observed between IGRA positivity and the diagnosis of TB in the meta-analysis. IGRA is widely used to detect TB infection and is generally negative in cases of NTM infection [30]. However, false-positive results have been reported, particularly in infections owing to certain NTM species, such as *M. kansasii *[31]. Therefore, IGRA results should be interpreted alongside other diagnostic indicators, including microbiological findings. Moreover, as IGRA cannot reliably distinguish between active tuberculosis and latent TB infection, additional clinical or laboratory data are necessary to support an accurate diagnosis of both diseases [32].

Beyond bronchiectasis and IGRA, recent efforts have focused on developing artificial intelligence (AI)-based approaches to analyze chest computed tomography images for automatic differentiation between pulmonary TB and NTM-PD, aiming to improve diagnostic accuracy. In a previous study involving 120 patients each diagnosed with pulmonary TB and NTM-PD, features such as tree-in-bud appearance and lymph node enlargement were identified as useful for differentiation, and AI-assisted interpretation enhanced diagnostic accuracy among physicians [33]. Furthermore, adjunctive diagnostic tools using biomarkers - such as lipoarabinomannan in blood or urine and the *M. tuberculosis *Ag85 complex - have been reported for the diagnosis of active TB. These tools are further expected to improve diagnostic precision [34].

This study has several limitations. First, many of the included studies used a retrospective design, which raises the possibility of bias. According to the QUADAS-2 tool, a high risk of bias was present in patient selection, potentially contributing to the observed heterogeneity. Second, most studies were conducted in Asia, raising concerns about regional variation. For example, the predominant causative species of NTM-PD vary by region [35], and this study was unable to evaluate how such differences might affect the differential diagnosis from pulmonary TB. Third, the number of studies assessing bronchiectasis and IGRA was limited, and the possibility of publication bias could not be ruled out. Therefore, further large-scale studies are required to validate these findings.

## Conclusions

In conclusion, this study suggests that the presence or absence of bronchiectasis is particularly useful for differentiating pulmonary TB from NTM-PD, which may facilitate earlier and more accurate diagnosis. However, because of interstudy heterogeneity and potential biases, further large-scale prospective studies are warranted to confirm these observations and improve diagnostic confidence in clinical settings.
